# Supplementary material for: Maximizing Collagen Yield from Underutilized Lumpfish (Cyclopterus lumpus) Skins by Optimizing Pre-Cleaning and Extraction Methods
Source: Mar Drugs. 2024 Nov 22;22(12):525. doi: 10.3390/md22120525 (PMC11678093; doi:10.3390/md22120525)
Supplement: Supplementary file 1 [file marinedrugs-22-00525-s001.zip › marinedrugs-3296651-supplementary.pdf]

Maximizing Collagen Yield from Underutilized Lumpfish (*Cyclopterus lumpus*) Skin by Optimizing Pre-Cleaning and Extraction Methods

Judith Maria Scheja, Jens Jakob Sigurðarson, Halldór Gunnar Ólafsson and Hjörleifur Einarsson

Supplementary Tables

Table S1: ANOVA of the main effects on the dependent variable protein content measured in the NaOH filtrate

	Df	Sum square	Mean square	F	p
Time	1	0.2141	0.2142	1.720e+30	< 2e-16
Concentration	1	0.0005	0.0005	4.277e+27	< 2e-16
Temperature	1	1.0294	1.0294	8.265e+30	< 2e-16
Ratio	1	0.9554	0.9554	7.670e+30	< 2e-16
Protein	1	0.6791	0.6791	5.452e+30	< 2e-16
Residuals	10	0.0000	0.0000		

Table S2: ANOVA of the main effects and 2-factor interactions on the dependent variable hydroxyproline content measured in the NaOH filtrate

	Df	Sum square	Mean square	F	p
Concentration	1	2384	2384	38.125	6.21e-06
Ratio	1	354	354	5.653	0.02808
Temperature	1	11925	11925	190.679	2.34e-11
Duration	1	1639	1639	26.202	6.10e-05
Concentration:ratio	1	51	51	0.810	0.37938
Concentration:temperature	1	1600	1600	25.576	7.00e-05
Concentration:duration	1	687	687	10.988	0.00364
Ratio:temperature	1	448	448	7.160	0.01495
Ratio:duration	1	31	31	0.496	0.48966
Temperature:duration	1	1645	1645	26.299	5.97e-05
Residuals	19	1188	63		
